# Microbiota and antibiotic exposure in sarcoidosis

**DOI:** 10.3389/fimmu.2025.1737273

**Published:** 2025-12-17

**Authors:** Claudio Ucciferri, Livia Moffa, Claudio Tana

**Affiliations:** 1 Clinic of Infectious Diseases - Department of Medicine and Science of Aging, “G. d’Annunzio” University Chieti, Pescara, Italy; 2 Department of Medicine, University of Perugia, Perugia, Italy; 3 Internal Medicine Unit, Eastern Hospital, Azienda Sanitaria Locale (ASL), Taranto, Italy

**Keywords:** sarcoidosis, microbiota dysbiosis, antibiotic exposure, immune regulation, lung–gut axis

## Abstract

**Background:**

Sarcoidosis is a multisystem granulomatous disorder characterized by excessive immune activation in genetically predisposed individuals. Despite decades of investigation, its etiology remains unresolved. Emerging evidence indicates that disruptions in host–microbiota homeostasis may contribute to immune dysregulation and disease persistence, challenging the traditional view of sarcoidosis as a purely immune-mediated condition.

**Perspective:**

Recent findings have revealed alterations in both respiratory and intestinal microbiota among patients with sarcoidosis, suggesting that microbial dysbiosis may influence T-cell polarization and granulomatous inflammation. Antibiotic exposure, particularly to macrolides and tetracyclines, represents a largely overlooked modifier that may alter this equilibrium through combined microbiota-dependent and immunomodulatory mechanisms. Given the frequent use of antibiotics in these patients for respiratory infections or off-label anti-inflammatory purposes, understanding their effect on microbial diversity, immune signaling, and disease course warrants systematic evaluation.

**Translational Outlook:**

We propose an integrative framework combining microbiota profiling, immune phenotyping, and therapeutic exposure to delineate microbiota–immunity interactions in sarcoidosis. Multi-omics strategies, supported by advanced computational and network-based analyses, could uncover microbe–immune signatures predictive of chronicity or treatment response. Defining how antibiotics shape these interactions may provide a foundation for microbiota-informed, immune-targeted, and ultimately personalized interventions.

**Conclusion:**

Deciphering the interplay between microbiota composition, antibiotic exposure, and immune regulation has the potential to reshape our understanding of sarcoidosis pathogenesis and to guide the development of precision-based therapeutic strategies.

## 1 Introduction

Sarcoidosis is a systemic granulomatous disorder of unknown etiology, characterized by non-caseating granulomas primarily affecting the lungs and intrathoracic lymph nodes (1). Despite decades of investigation, the initiating antigens and regulatory mechanisms underlying granuloma formation remain poorly understood (2). The disease is thought to emerge from an exaggerated cellular immune response to poorly cleared antigens in genetically predisposed individuals, leading to sustained Th1/Th17 activation and impaired immune resolution (3, 4). However, the nature of infectious, environmental, or self-derived antigens has long remained elusive.

The concept of host–microbiota homeostasis has reshaped our view of immune-mediated diseases, as microbial imbalance is increasingly linked to altered tolerance and inflammation in disorders such as inflammatory bowel disease, rheumatoid arthritis and hypersensitivity pneumonia (5). In this context, growing evidence suggests that sarcoidosis may also involve a breakdown in host–microbiota balance, rather than a single microbial trigger.

Preliminary studies suggest alterations in the respiratory and intestinal microbiota of patients with sarcoidosis, with taxa such as *Propionibacterium acnes* and *Mycobacterium* spp. detected within granulomatous lesions. These findings support the hypothesis that the microbiota may act as a context-dependent immunological amplifier in genetically susceptible hosts (6, 7).

An additional, largely overlooked variable is antibiotic exposure, which can profoundly reshape microbial ecosystems. Antibiotics such as macrolides and tetracyclines are frequently administered to sarcoidosis patients for coexisting respiratory infections or off-label anti-inflammatory effects (8). Beyond their antimicrobial action, these agents possess immunomodulatory properties, including the inhibition of cytokine release, oxidative stress, and metalloproteinase activity. Consequently, their use may alter the microbiota–immune equilibrium, either dampening or perpetuating granulomatous inflammation (9).

This Perspective aims to discuss the potential interplay between microbiota composition, antibiotic exposure, and immune dysregulation in sarcoidosis. By integrating current evidence from microbiome research, immunology, and clinical pharmacology, we propose a conceptual framework in which microbial community dynamics and antibiotic-modulated immune responses may jointly influence disease persistence or resolution. Understanding this tripartite relationship could offer novel insights into sarcoidosis pathogenesis and inform future microbiota-targeted or immune-based therapeutic strategies.

## 2 The microbiota–immune interface in sarcoidosis

The human microbiota, spanning the respiratory and gastrointestinal tracts, plays a central role in maintaining immune homeostasis and mucosal integrity (10). Increasing evidence also suggests that microbial imbalance may contribute not only to immune-mediated and metabolic disorders but also to neuroinflammatory and cognitive dysfunction through the gut–brain axis (11). Far from being sterile, the lung hosts a low-biomass but immunologically active microbial community that interacts continuously with epithelial and immune cells through pattern recognition receptors (PRRs), including Toll-like receptors (TLRs) and NOD-like receptors (12). This microbial dialogue contributes to immune tolerance by modulating dendritic-cell maturation, macrophage polarization, and the balance between effector and regulatory T cells (Th1/Th17 versus Treg) (12). When this equilibrium is disrupted through infection, environmental exposure, or antibiotic use, the immune system may shift toward chronic activation, tissue injury, and granuloma formation (12).

In sarcoidosis, immune dysregulation is dominated by Th1 and Th17 activation and inadequate regulatory T-cell control, resulting in sustained cytokine release (e.g., IFN-γ, TNF-α, IL-17) and macrophage aggregation into non-caseating granulomas (13). Microbiota can influence these pathways through the production of microbial metabolites such as short-chain fatty acids (SCFAs), tryptophan catabolites, and bile acid derivatives. SCFAs, particularly butyrate and propionate, promote Treg differentiation and suppress pro-inflammatory cytokines (14). SCFAs depletion, observed in several immune-mediated diseases, may facilitate the persistence of Th1/Th17-driven inflammation like that seen in sarcoidosis (15, 16).

The gut–lung axis provides a key framework for understanding these interactions. Microbial products and metabolites originating from the gut reach the systemic circulation, influencing distant mucosal sites such as the lung. Alterations in gut microbial composition can thus have immunological repercussions beyond the intestine (17). In diseases such as asthma, COPD, and idiopathic pulmonary fibrosis, gut dysbiosis has been associated with heightened systemic inflammation and altered immune cell trafficking (18). Although data in sarcoidosis remain limited, pilot studies have reported reduced bacterial diversity and an imbalance between anti- and pro-inflammatory taxa in stool samples of affected individuals, supporting a systemic dimension of microbial imbalance (19).

At the local level, bronchoalveolar lavage (BAL) analyses have shown decreased microbial diversity in sarcoidosis compared with healthy controls, with enrichment of genera such as *Atopobium* and *Fusobacterium* (20). These microorganisms are capable of engaging TLR2 and TLR4 signaling pathways, promoting macrophage activation and the release of TNF-α and IL-1β—cytokines essential for granuloma formation (21, 22). In summary, several mechanisms may link microbial imbalance to the dysregulated immunity of sarcoidosis. Reduced levels of key microbial metabolites such as SCFAs, indoles, and bile acid derivatives, may weaken Treg induction, favor Th1/Th17 polarization, and sustain macrophage activation, thereby promoting granuloma persistence (14, 15, 21). Dysbiosis may also compromise epithelial barrier integrity and increase exposure to microbial ligands that activate PRR/TLR pathways, amplifying NF-κB– and STAT-mediated cytokine responses (21).

Whether dysbiosis represents a primary contributor to immune imbalance or a secondary effect of inflammation or treatment remains debated (23). Some findings suggest upstream microbial influences, while others indicate that chronic inflammation and therapy can themselves reshape microbial communities (15). Recognizing both possibilities highlights the need for longitudinal, treatment-naïve studies.

## 3 Antibiotic exposure as a modulator of microbiota and immune homeostasis

Antibiotics represent one of the most powerful but also most disruptive forces acting on the human microbiota. While indispensable for controlling infection, they can profoundly alter microbial diversity, metabolic capacity, and ecological resilience (24). In the respiratory and gastrointestinal tracts, repeated or prolonged antibiotic exposure reduces microbial richness, favors overgrowth of opportunistic species, and alters metabolite profiles, and these changes can persist for months after treatment cessation (25). These perturbations are particularly relevant in immune-mediated diseases such as sarcoidosis, where immune balance and microbial signals are tightly interwoven. It is important to distinguish microbiota-dependent pathways such as SCFA- and indole-mediated modulation of pulmonary immunity, from the direct immunomodulatory effects of antibiotics. Current evidence often overlaps these two levels, underscoring the need for studies designed to separate microbial-mediated mechanisms from the direct anti-inflammatory actions of macrolides and tetracyclines (26).

Sarcoidosis patients often receive antibiotics for concomitant respiratory infections, for suspected infectious exacerbations, or, less frequently, for off-label anti-inflammatory purposes (26). Among these, macrolides (e.g., azithromycin, clarithromycin) and tetracyclines (e.g., doxycycline, minocycline) are the most frequently used and have well-recognized immunomodulatory effects independent of their antibacterial activity (26). Macrolides down-regulate neutrophil chemotaxis, IL-8 and TNF-α production, and matrix metalloproteinase release, while promoting macrophage polarization toward a reparative M2 phenotype (26). Tetracyclines exert antioxidant and anti-apoptotic actions and can inhibit microglial and T-cell activation. Such properties have prompted their evaluation in chronic inflammatory lung diseases (27, 28). However, these same agents can markedly alter both lung and gut microbiota, reducing commensal taxa such as *Bifidobacterium* and *Lactobacillus* and increasing the prevalence of antibiotic-resistant or pro-inflammatory organisms (29).

The dual impact (antimicrobial and immunoregulatory) of antibiotics raises a key question for sarcoidosis: do these drugs transiently suppress inflammation or do they inadvertently reshape microbial ecosystems in ways that sustain disease activity? Evidence from other conditions supports both possibilities. In COPD and bronchiectasis, long-term macrolide therapy decreases exacerbation rates but could be associated with persistent loss of microbial diversity and enrichment of resistant strains (30). In inflammatory bowel disease, antibiotic exposure has been associated with altered SCFAs production and dysbiosis (31). Translating these insights to sarcoidosis, one could hypothesize that antibiotic-induced microbial imbalance reduces protective metabolites and disrupts mucosal tolerance, thereby amplifying immune polarization and granulomatous persistence.

Furthermore, antibiotics may modify antigen availability and processing. By altering the airway microbiome, they could reduce exposure to certain microbial antigens while enhancing others, potentially shifting antigen-presenting-cell activation and cytokine profiles (32). This dynamic may help explain clinical observations where some patients experience transient improvement during antibiotic therapy followed by relapse once treatment ends. A summary of the main mechanisms linking microbiota composition, immune regulation, and antibiotic exposure in sarcoidosis is provided in **Table 1**.

**Table 1:** Microbiota, immune regulation, and antibiotic exposure in sarcoidosis: main mechanisms and implications.

Key mechanisms	Potential impact on sarcoidosis	Representative evidence/remarks
Reduced microbial diversity in BAL with enrichment of Streptococcus, Veillonella, and Cutibacterium	Activation of TLR-mediated pathways, macrophage stimulation, and Th1/Th17 polarization sustaining granulomatous inflammation	BAL metagenomic and culture-based studies
Loss of SCFA-producing intestinal taxa and enrichment of pro-inflammatory species	Impaired Treg induction and systemic immune priming through the gut–lung axis	Pilot cohorts and parallels with IBD and COPD
Reduced production of microbial metabolites (SCFAs, tryptophan and bile acid derivatives)	Decreased immunoregulatory signaling and persistence of pro-inflammatory cytokine loops	Mechanistic data from autoimmune and granulomatous diseases
Exposure to macrolides and tetracyclines with dual antimicrobial and immunomodulatory effects	Transient control of inflammation but potential long-term dysbiosis and immune imbalance	Clinical observations in COPD and sarcoidosis case series
Dysregulated Th1/Th17–Treg balance and altered macrophage activation in a genetically susceptible host	Maintenance of chronic granulomatous inflammation and disease persistence	Core immunopathogenic hallmark of sarcoidosis
Integration of microbiota data, immune phenotyping, and therapeutic exposure through multi-omics and AI tools	Identification of microbe–immune signatures and novel microbiota-targeted therapeutic strategies	Proposed systems and translational framework

**Table 1**. Summary of key mechanisms linking microbiota composition, immune regulation, and antibiotic exposure in sarcoidosis. BAL, broncoalveolar lavage; SCFAs , short-chain fatty acids; Th , Thelper; ,IBD , Inflammatory Bowel Disease; COPD , Chronic Obstructive Pulmonary Disease.

Despite the plausible biological relevance, systematic data on antibiotic exposure in sarcoidosis are almost entirely lacking. Clinical studies rarely document the duration, class, or timing of antibiotic use, and microbiome research seldom controls for this variable. Future investigations should therefore include detailed antibiotic histories and integrate them into multi-omics analyses to clarify how therapeutic or incidental antibiotic courses influence microbial and immune trajectories.

Recognizing antibiotics not merely as confounders but as potential modulators of host–microbiota interactions could refine our interpretation of existing data and guide new strategies. Carefully designed longitudinal studies and experimental models are needed to determine whether modulating microbial communities after antibiotic exposure through diet, probiotics, or microbiota-targeted interventions, can restore immune equilibrium and favor disease resolution in sarcoidosis. At the same time, although macrolides may provide short-term anti-inflammatory benefits, their use presents a clinical dilemma, as they can also induce long-term dysbiosis and antimicrobial resistance. Balancing these immediate effects against the potential disruption of host–microbiota homeostasis is therefore essential.

## 4 A systems and translational framework for future research

Understanding the interplay among microbiota composition, immune pathways, and therapeutic exposure in sarcoidosis requires a systems-level approach. Traditional single-modality studies which focus exclusively on microbial composition, immune profiling, or clinical outcomes, have provided valuable but fragmented insights (33). To move beyond descriptive observations, future research must integrate these domains within a unified analytical framework capable of capturing the complexity of host–microbe–therapy interactions (34).

A multi-omics strategy represents a logical next step. High-throughput sequencing of bacterial 16S rRNA and shotgun metagenomics can delineate taxonomic and functional changes in both the gut and lung microbiota, while metabolomics and transcriptomics can identify signaling metabolites and immune pathways linked to disease activity (35). Integration of these datasets through computational network modeling and machine learning allows the identification of microbe–immune signatures predictive of clinical phenotypes such as chronic versus self-limiting disease (36). Artificial intelligence (AI) tools, including manifold alignment and multi-modal representation learning, can harmonize heterogeneous data, uncover latent biological structures, and generate predictive models of immune dysregulation (37).

Crucially, this systems approach should incorporate detailed therapeutic metadata, particularly antibiotic exposure. Recording the timing, duration, and class of antibiotics is essential to discern whether microbial shifts represent disease-driven dysbiosis or treatment-induced perturbations. Linking these variables with immune phenotypes such as Th1/Th17 polarization, Treg deficiency, and macrophage activation states, may reveal causal relationships obscured in previous cross-sectional analyses. Such integration could also clarify whether antibiotic-modulated changes in microbial metabolites (e.g., SCFAs, tryptophan derivatives) directly influence cytokine signaling or granuloma dynamics (38).

From a translational standpoint, data-driven stratification of patients according to microbiota and immune profiles could lead to more precise phenotyping and risk prediction. Identifying microbiota-associated immune signatures might help distinguish individuals likely to experience disease remission from those at risk of chronic or relapsing forms (39). These insights could, in turn, inform microbiota-targeted interventions, such as probiotic supplementation, dietary modulation, or post-antibiotic microbiome restoration, potentially used alongside immunomodulatory therapies.

Collaboration between clinical centers, microbiologists, immunologists, and computational scientists will be pivotal for achieving sufficient sample sizes and analytical depth (36). Establishing standardized multicenter biobanks for stool, BAL, and serum samples, together with harmonized clinical metadata, would facilitate reproducibility and enable cross-validation of findings (34). Incorporating in silico modeling and AI-based analytics can transform these complex datasets into actionable biological hypotheses (34).

Ultimately, adopting a systems and translational framework will allow the field to progress from associative observations to mechanistic understanding. Notably, direct clinical evidence on how antibiotics modulate the microbiota specifically in sarcoidosis is almost entirely lacking, and current biological inferences rely largely on data derived from COPD, IBD, and other inflammatory conditions (40, 41). Prospective, longitudinal cohort studies with pre- and post-antibiotic sampling, combined with standardized multi-omics profiling, are likely the most suitable designs to clarify causal relationships (37, 42). Such approaches should also systematically account for key confounders, particularly corticosteroids and other immunosuppressants, which can independently modify both immune phenotypes and microbial communities (37). Incorporating these methodological elements will strengthen the feasibility and interpretability of future AI-driven analyses (37, 42).

Integrating microbiome science, immunology, and computational modeling could redefine sarcoidosis not merely as an enigmatic granulomatous disorder but as a dysregulated network disease arising from the disruption of host–microbiota equilibrium under therapeutic and environmental pressures.

## 5 Conclusions

Sarcoidosis remains an enigmatic immune-mediated disorder shaped by the interplay between genetic susceptibility, environmental exposures, and dysregulated immunity. Emerging evidence that the lung and gut microbiota influence immune homeostasis adds a new dimension to understanding disease persistence. Altered microbial diversity and the frequent, often overlooked use of antibiotics suggest that disruptions of host–microbiota balance may contribute more substantially to the inflammatory environment of sarcoidosis than previously recognized.

This perspective shifts the focus from a single infectious trigger to an integrated model in which microbial communities, immune networks, and therapeutic exposures interact dynamically. Future studies should therefore include detailed antibiotic histories, longitudinal microbiota profiling, and immune phenotyping to help distinguish cause from consequence.

Although clinical translation remains preliminary, these insights may ultimately support better patient stratification, guide post-antibiotic microbiome recovery, and inform complementary microbiota-oriented interventions. Reframing sarcoidosis as a disorder of disrupted host–microbiota homeostasis may pave the way toward precision immunology, with treatments tailored not only to immune phenotypes but also to the microbial ecosystems that shape them.

## Data Availability

The original contributions presented in the study are included in the article/supplementary material. Further inquiries can be directed to the corresponding author/s.
