# Correction: Microbiota and antibiotic exposure in sarcoidosis

**DOI:** 10.3389/fimmu.2026.1782967

**Published:** 2026-01-22

**Authors:** Claudio Ucciferri, Livia Moffa, Claudio Tana

**Affiliations:** 1 Clinic of Infectious Diseases - Department of Medicine and Science of Aging, “G. d’Annunzio” University Chieti, Pescara, Italy; 2 Department of Medicine, University of Perugia, Perugia, Italy; 3 Internal Medicine Unit, Eastern Hospital, Azienda Sanitaria Locale (ASL), Taranto, Italy

**Keywords:** sarcoidosis, microbiota dysbiosis, antibiotic exposure, immune regulation, lung–gut axis

Affiliation “Department of Medicine, University of Perugia, Perugia, Italy” was omitted for author Claudio Ucciferri. This affiliation has now been added for author Claudio Ucciferri. 

The original version of this article has been updated.

